# Efficacy of a Wearable Activity Tracker With Step-by-Step Goal-Setting on Older Adults’ Physical Activity and Sarcopenia Indicators: Clustered Trial

**DOI:** 10.2196/60183

**Published:** 2024-11-01

**Authors:** Mu-Hsing Ho, Chi-Yuan Peng, Yung Liao, Hsin-Yen Yen

**Affiliations:** 1 School of Nursing Li Ka Shing Faculty of Medicine The University of Hong Kong Pokfulam China (Hong Kong); 2 School of Gerontology and Long-term Care College of Nursing Taipei Medical University Taipei City Taiwan; 3 Graduate Institute of Sport, Leisure, and Hospitality Management National Taiwan Normal University Taipei Taiwan

**Keywords:** behavioral change technique, chronic disease prevention, health promotion, mHealth, sedentary behavior, smartwatch

## Abstract

**Background:**

Smart wearable technology has potential benefits for promoting physical activity and preventing sarcopenia.

**Objective:**

The purpose of this study was to explore the efficacy of a wearable activity tracker with 2-stage goal-setting for daily steps on older adults’ physical activity and sarcopenia indicators.

**Methods:**

The study used a clustered trial design and was conducted in March to June 2022. Participants were community-dwelling adults older than 60 years who were recruited from 4 community centers in Taipei City. The intervention was designed with 2-stage goals set to 5000 steps/day in the first 4 weeks and 7500 steps/day in the final 4 weeks while wearing a commercial wearable activity tracker. Data were collected by self-reported questionnaires, a body composition analyzer, a handle grip tester, and 5 sit-to-stand tests.

**Results:**

All 27 participants in the experimental group and 31 participants in the control group completed the 8-week intervention. Total and light-intensity physical activities, skeletal muscle index, and muscle strength increased, while sedentary time, BMI, and the waist circumference of participants decreased in the experimental group, with significant group-by-time interactions compared to the control group.

**Conclusions:**

A wearable activity tracker with gradual goal-setting is an efficient approach to improve older adults’ physical activity and sarcopenia indicators. Smart wearable products with behavioral change techniques are recommended to prevent sarcopenia in older adult populations.

## Introduction

Sarcopenia is an urgent health challenge in later life. Older adults with sarcopenia have increased risks of falls, fractures, and disabilities; a poor quality of life; functional declines; and increased mortality [1]. Muscle mass, muscle strength, and physical performance are 3 indicators of sarcopenia [2]. Muscle mass of adults may decrease 8% per decade after the age of 40 years, and muscle mass rapidly decreases by 10% to 15% per decade after the age of 70 years [3]. Muscle strength decreases by 10% to 15% per decade after the age of 40 years, and by 25% to 40% per decade after the age of 70 years [4]. Physical functions decline more rapidly, at 1.5% per year between the ages of 50 and 60 years and 3% per year thereafter [5]. The prevalence rate of sarcopenia was estimated to be 10% in both older men and older women [6].

Engaging in physical activity (PA) is one preventive strategy to slow the development of sarcopenia in older adult populations [7,8]. Sufficient PA can improve older adults’ gait speed, balance, physical functions, coordination, and activities of daily living [9]. Sufficient PA is generally defined as engaging in PA to meet recommended guidelines for maintaining health and preventing disease, such as those proposed by the World Health Organization, American College of Sports Medicine, and other health care institutions [10], or achieving a minimum of 600 metabolic equivalents of task (MET)–minutes per week [11]. PA promotion and structured exercise training programs were found to improve muscle mass, grip and leg muscle strength, and physical performance in older adult populations both with and without sarcopenia [12,13].

Walking is a key way to stay active and expend energy in daily life. It is an easy, feasible, and convenient option for older adults of various ages and health conditions [14]. Older adults are recommended to walk at least 7000 steps/day, which is equivalent to at least 30 minutes/day (or 150 minutes/week) of PA [15]. A previous prospective cohort study found that middle-aged adults who walked more than 7000 steps/day had a 50% to 70% lower risk of death [16]. For older adults with an inactive and sedentary lifestyle, at least 4600 to 5500 steps per day are recommended to reduce the risk of developing chronic diseases. An increase of 1000 steps/day revealed additional health benefits [17]. A previous investigation of an older population aged over 60 years indicated that increasing walking may decrease the odds of sarcopenia. The link between sarcopenia and PA was particularly evident in those who walked [18].

Smart wearable technology is an innovative approach to modify PA in daily life to prevent sarcopenia. Commercial wearable activity trackers (WATs) have features that help users stay motivated and on track with their fitness goals. These devices continually monitor PA levels, provide real-time feedback, set achievable goals, offer reminders, and even encourage social interactions and competition [19-21]. For instance, a WAT allows users to effortlessly track their daily steps, monitor heart rate, and measure energy expenditure as part of their routine. It can also send gentle reminders to stand up and break up sedentary habits, while providing an easy way to review progress toward their PA goals [22,23]. The combination of WATs and smartphone apps can enhance the effects of PA interventions [24]. However, the validity of WATs as PA-measuring tools, using sensors like accelerometers and GPS to track movement, steps, and heart rate, is still a problem. The accuracy can vary by device and activity type and depends on consistent use. Their accuracy may be less reliable for PA like cycling or strength training compared to walking [25].

Older adults are increasingly embracing WATs due to their potential health benefits and personalized features, such as comfort, sleek designs, and user-friendly interfaces [26,27]. A key factor in the success of WATs is self-efficacy—the belief in one’s own ability to succeed. This is essential for using WATs to enhance and maintain PA levels [28]. For older adults, low self-efficacy can diminish motivation, making it harder to maintain healthy habits [29]. WATs support users by helping them gradually achieve short-, medium-, and long-term goals through consistent encouragement and reminders, rather than pushing them toward unrealistic targets [30]. By providing behavioral change techniques, WATs effectively increase older adults’ self-efficacy, awareness, and proactive engagement in PA and health self-management [23].

Previous experimental studies suggested that wearable-based interventions, including WATs, pedometers, and other fitness trackers, have potential benefits in improving PA and modifying healthy behaviors [22,24,31,32]. Also, PA had positive impacts on reducing the risks and incidence of sarcopenia [8,33] and improving muscle mass, muscle strength, and physical performance in older community-dwelling adults [7,34].

In this study, we developed an innovative wearable-based intervention with behavioral change technologies. The intervention was designed as 2-stage goal-setting for daily steps via a commercial WAT. A nutritional health educational program that focused on healthy eating was also conducted in both the control and experimental groups. The study purpose was to explore the efficacy and feasibility of an intervention among community-dwelling older adults and investigate PA and sarcopenia indicators. Outcomes included multi-intensity PA, sedentary time, waist circumference (WC), BMI, muscle mass, muscle strength, and physical performance.

## Methods

### Study Design

This study was a clustered trial conducted in March to June 2022. Participants were recruited from 4 community centers in Taipei City. Of these 4 centers, 2 were assigned to either an experimental group or a control group by a draw. Participants were not aware of the other groups because the community centers were in different locations.

### Participants

Participants were community-dwelling older adults. All community centers funded as part of the local long-term care policy serve as primary care facilities. These centers are designated to assist healthy older adults who live independently, have no disabilities, can move about without physical limitations, and have chronic conditions that do not currently require urgent medical attention.

The inclusion criteria were (1) being an adult older than 60 years, (2) having a smartphone that can download the app connected to the WAT, (3) being able to learn how to use the WAT, and (4) having used no device to monitor daily steps in the previous 6 months. The exclusion criteria were (1) having any disease or cancer that was currently under medical care (ie, surgery, chemotherapy, or hemodialysis); (2) having any diagnosed disability (ie, dementia or hearing or visual impairment), (3) being unable to walk on one’s own; and (4) having any electronic device inside one’s body. The minimum sample size was precalculated by medium effect sizes via G*power (Universität Düsseldorf) with an added 20% attrition rate, resulting in a sample size of 41. In total, 70 participants were recruited from 4 clusters; 58 participants were assessed as meeting the criteria. Finally, 27 participants were allocated to the experimental group, and another 31 participants were allocated to the control group.

### The Intervention

The intervention used a WAT connected to a smartphone app, which was set to progressively increase daily step goals over an 8-week period. Participants in the experimental group were required to wear a commercial WAT, the Asus VivoWatch BP (HC-A04; Asus), on their wrist. Participants were carefully taught how to use the WAT and the smartphone app, and they were introduced to all features before the intervention. Features included tracking of daily steps, walking distance, heart rate, calories burned, exercise duration, and notifications of long sedentary time. By connecting to the smartphone app, the user could access integrated information about tracking results and gain personalized health advice. Participants were encouraged to use the WAT during their waking time and only take it off to charge during sleep and when bathing every day. Participants were allowed to freely use the smartphone app without specific restrictions. The social media account of the research team was provided in case participants had any difficulty using the WAT. They could contact the research team at any time to resolve problems.

The 2-stage goals were 5000 steps/day in the first month (weeks 1-4) and 7500 steps/day in the second month (weeks 5-8). The research team set the step goals on their smartphones. When a participant reached the target number of daily steps during the wearing time, the WAT would vibrate and display a “trophy” image to inform them. Achievements were recorded on the smartphone app, which allowed the older adults to check them at any time. Reminders and achievements can encourage older adults to keep pursuing their goals each day through self-efficacy. In addition, once a week, participants received group-based nutritional health education about healthy eating. During the weekly group session, a research team member checked use of the WAT, and participants could ask for help with any difficulties using the WAT to resolve the problem. Participants who mentioned having lower wearing time were reminded to use the WAT more frequently.

Participants in the control group received the same weekly nutritional health education during the 8 weeks of the study.

### Outcome Measures

Data were collected twice, at the pretest and postintervention stages, by a trained master’s degree student. Participant characteristics were collected before the intervention, including age, sex, marital status, educational level, and presence of chronic diseases (yes/no).

### PA and Sedentary Time

Subjective data were collected by structured questionnaires via one-on-one interviews. The International Physical Activity Questionnaire–Short Form (IPAQ-SF) was used to assess participants’ PA and sedentary time in the past week through 7 items. The IPAQ-SF has good validity and internal consistency [11]. Daily sedentary hours and MET-min/week of light-intensity PA (LPA), moderate-to-vigorous-intensity PA (MVPA), and total PA were calculated as outcomes.

### Sarcopenia Indicators, BMI, and WC

Sarcopenia indicators were measured according to the Asian Working Group for Sarcopenia [2]. BMI and skeletal muscle index (SMI) were measured with a body composition analyzer (Inbody 270; Inbody) using bioelectrical impedance analytical technology. The participant stepped barefoot onto the foot electrodes while gripping the hand electrodes. During this time, the participant stood still and kept their arms straight without touching for 1 minute. A higher score (kg/m^2^) for SMI indicates greater muscle mass. Muscle strength was assessed by a handle grip instrument (Tokyo Telecommunication Machine Corp). Participants were asked to stand, allowing the wrist and arm of the dominant hand to hang straight down. The individual squeezed the device with maximum effort for more than 3 seconds. This measurement was repeated 3 times. The highest value recorded from multiple trials was used as an indicator of muscle strength. Physical performance was measured by 5 sit-to-stand tests. The participant was asked to rise from a seated position in a chair to a full standing position and then sit back down with no assistance as quickly as possible 5 times. A timer measured the time required to complete the task. The total time taken to complete 5 repetitions was recorded. A longer time (in seconds) indicated worse physical performance. In addition, to measure WC, a flexible, nonstretchable tape was used. The tape was positioned horizontally around the abdomen, just above the belly button, between the bottom of the rib cage and the top of the hip bones. The measurement was taken after a normal exhalation.

### Statistical Analysis

Descriptive analyses included the frequency and percentage of participants’ characteristics and the mean and SD of baseline outcome scores. Chi-square tests and independent 2-tailed *t* tests were performed to compare participants’ characteristics and baseline outcome scores between the control and experimental groups. An analysis of covariance (ANCOVA) was performed to compare participants’ posttest scores between the 2 groups by controlling for their age and pretest scores. Paired 2-tailed *t* tests were performed to analyze differences in subjective and objective outcomes between the pretest and postintervention stages in each group with the mean difference (MD) and 95% CI. Cohen *d* and η^2^ were calculated for effect sizes. Generalized estimating equations (GEEs) were used to analyze group-by-time interactions on outcomes, which were adjusted for participants’ age and baseline scores in the pretest. After testing, all outcome values exhibited a normal distribution. SPSS (version 18.0; IBM Corp) was used for all statistical analyses.

### Ethical Considerations

Participants were required to sign an informed consent form agreeing to participate before entering the study. All participants’ data were handled with strict confidentiality and privacy throughout the storage and analysis processes. Data were anonymized and securely stored in compliance with applicable data protection regulations, ensuring that personal information was not accessible to unauthorized individuals. All participants were fully informed prior to the intervention that no financial compensation or material benefits would be provided upon its completion. Ethical approval for this study was obtained from the Taipei Medical University Joint Institutional Review Board (N202202031).

## Results

**Figure 1:**
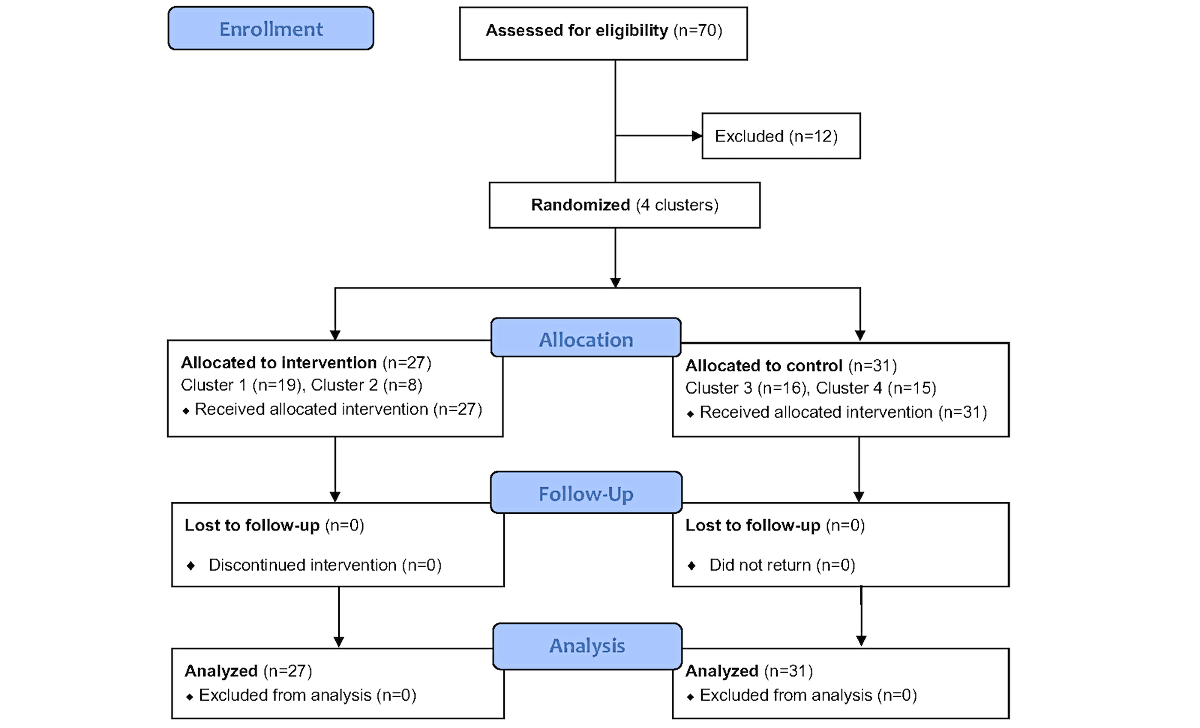
Consolidated Standards of Reporting Trials (CONSORT) flow diagram.

The study flow is illustrated in Figure 1. Participant characteristics and outcome scores at baseline are described in Tables 1 and 2. Sex, marital status, educational level, and the presence of chronic disease had no significant differences between the experimental and control groups. However, participants in the control group (mean age 74.26, SD 6.30 years) were significantly older than those in the experimental group (mean age 70.26, SD 4.72 years; *P*=.01). Participants’ sedentary time at baseline significantly differed between the 2 groups (*P*=.048). No other outcome score at baseline significantly differed between the 2 groups (*P*<.05). None of the participants dropped out of the study. All 27 participants in the experimental group and 31 participants in the control group completed the intervention and were included in subsequent analyses.

**Table 1:** Participant characteristics.

Characteristics			Experimental group (n=27)		Control group (n=31)		*P* value	
**Sex, n (%)**								.39
	Male	3 (11.11)		6 (19.35)				
	Female	24 (88.89)		25 (80.65)				
**Marital status, n (%)**								.36
	Married	17 (62.96)		23 (74.19)				
	Single, divorced, or widowed	10 (37.04)		8 (25.81)				
**Education, n (%)**								.13
	None to elementary school	7 (25.93)		14 (45.16)				
	High school or higher	20 (74.07)		17 (54.84)				
**Chronic diseases, n (%)**								.88
	No	11 (40.74)		12 (38.71)				
	Yes	16 (59.26)		19 (61.29)				
**Age (years), mean (SD)**			70.26 (4.72)		74.26 (6.30)		.01	

**Table 2:** Comparison of participant outcomes at the pre-and posttest stages between the groups.

Outcome				Experimental group			Control group			*P* value			Cohen *d*			η^2^	
**Pretest scores, mean (SD)**																	
	Waist circumference (cm)		85.35 (8.34)			84.47 (9.62)			.71			0.10			—^a^		
	BMI (kg/m^2^)		24.39 (2.71)			23.86 (3.18)			.50			0.18			—		
	**Physical activity**																
		Total (MET^b^-min/week)			1609.33 (1443.41)			1653.71 (1605.98)			.91			0.03	—		
		Moderate to vigorous (MET-min/week)			700.00 (841.77)			636.13 (888.41)			.78			0.07	—		
		Light intensity (MET-min/week)			909.33 (1034.58)			1016.94 (934.20)			.68			0.11	—		
		Sedentary time (h/day)			45.59 (8.18)			45.45 (4.66)			.048			0.02	—		
	**Sarcopenia indicators**																
		Skeletal muscle index (kg/m^2^)			6.20 (0.64)			5.99 (1.17)			.40			0.22	—		
		Muscle strength (kg)			22.69 (6.10)			20.26 (6.95)			.17			0.37	—		
		Physical performance (s)			7.97 (2.34)			9.18 (2.97)			.10			0.45	—		
**Posttest scores, mean (SE)**																	
	Waist circumference (cm)		84.74 (0.77)			85.88 (0.71)			.30			—			0.02		
	BMI (kg/m^2^)		24.06 (0.06)			24.49 (0.06)			<.001			—			0.31		
	**Physical activity**																
		Total (MET-min/week)			2056.65 (244.26)			1225.37 (227.00)			.02			—	0.10		
		Moderate to vigorous (MET-min/week)			258.19 (104.67)			394.48 (97.27)			.36			—	0.02		
		Light intensity (MET-min/week)			1777.87 (201.38)			848.83 (187.14)			.002			—	0.17		
		Sedentary time (h/day)			5.53 (0.41)			6.69 (0.37)			.06			—	0.07		
	**Sarcopenia indicators**																
		Skeletal muscle index (kg/m^2^)			6.13 (0.04)			6.24 (0.04)			.06			—	0.07		
		Muscle strength (kg)			22.31 (0.44)			20.96 (0.41)			.03			—	0.08		
		Physical performance (s)			7.00 (0.28)			8.07 (0.26)			.01			—	0.12		

^a^Not applicable.

^b^MET: metabolic equivalent of task.

Table 2 also shows comparisons of participants’ outcomes at the pre- and posttests between the 2 groups. After adjusting for age and pretest scores, participants in the experimental group had a significantly lower BMI, higher total PA and LPA, and better muscle strength and physical performance than those in the control group. WC, MVPA, sedentary time, and SMI did not show a significant difference between the 2 groups at the posttest.

**Figure 2:**
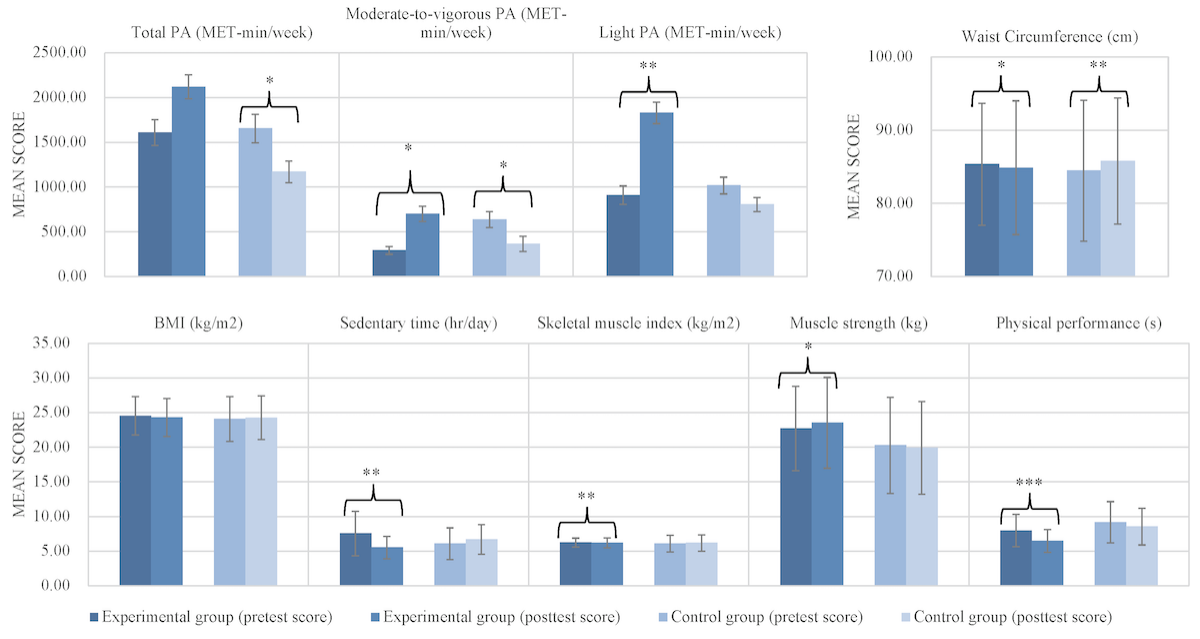
Results of outcome changes within each group. MET: metabolic equivalent of task; PA: physical activity. *P<.05, **P<.01, ***P<.001.

Paired *t* test and GEE results are presented in Figure 2 and Table 3. After the 8-week intervention, the total PA (MD 510.96 MET-min/week), LPA (MD 919.11 MET-min/week), SMI (MD 0.09 kg/m^2^), and muscle strength (MD 0.84 kg) of participants in the experimental group had increased, while those of participants in the control group had decreased, with significant group-by-time interactions (*P*=.03, *P*=.002, *P*<.001, and *P*<.001, respectively). Sedentary time (MD –2.02 h/day), BMI (MD –0.21 kg/m^2^), and WC (MD –0.48 cm) decreased for participants in the experimental group, while they increased in the control group, with significant group-by-time interactions (*P*=.001, *P*=.04, and *P*<.001, respectively). The MVPA of participants in both groups decreased without a significant group-by-time interaction (*P*=.41). Physical performance of participants in the experimental group (MD –1.50 s) improved compared to the control group (MD –0.64 s), with a significant group-by-time interaction (*P*=.04).

Within the experimental group, neither participants’ BMI nor total PA showed a significant change after the intervention. Within the control group, participants’ WC increased, and MVPA and total PA decreased after the 8 weeks. No other outcomes showed a significant change after the 8 weeks, including BMI, LPA, sedentary time, and all sarcopenia indicators.

**Table 3:** Differences within and between the experimental and control groups. Generalized estimating equations were adjusted for participant age and pretest scores; Cohen *d* was used for effect size.

Outcome	Experimental group				Control group				Group × time interaction (*P* value)
	MD^a^ (95% CI)	*P* value	Cohen *d*	MD (95% CI)		*P* value	Cohen *d*		
Waist circumference (cm)	–0.21 (–0.38 to 0.04)	.02	0.14	0.17 (0.08 to 0.27)		.001	0.30	<.001	
BMI (kg/m^2^)	–0.48 (–1.87 to 0.90)	.48	0.50	1.30 (–0.30 to 2.89)		.11	0.73	.04	
Total PA^b^ (MET^c^-min/week)	510.96 (–297.50 to 1319.43)	.21	0.25	–483.77 (–967.81 to 0.26)		.049	0.37	.03	
MVPA^d^ (MET-min/week)	–408.15 (–741.53 to 74.77)	.02	0.48	–270.97 (–432.29 to 109.64)		.002	0.62	.41	
LPA^e^ (MET–min/week)	919.11 (244.05 to 1594.17)	.01	0.54	–212.16 (–613.22 to 188.90)		.29	0.19	.002	
Sedentary time (h/day)	–2.02 (–3.51 to 0.53)	.01	0.54	0.63 (–0.29 to 1.55)		.17	0.25	.001	
Skeletal muscle index (kg/m^2^)	0.09 (0.02–0.16)	.01	0.10	–0.02 (–0.10 to 0.06)		.61	0.54	<.001	
Muscle strength (kg)	0.84 (0.03 to 1.64)	.04	0.41	–0.36 (–1.20 to 0.48)		.39	0.16	<.001	
Physical performance (s)	–1.50 (–2.14 to 0.86)	<.001	0.92	–0.64 (–1.29 to 0.00)		.05	0.37	.04	

^a^MD: mean difference between pre- and posttest score.

^b^PA: physical activity.

^c^MET: metabolic equivalent of task.

^d^MVPA: moderate-to-vigorous-intensity physical activity.

^e^LPA: light-intensity physical activity.

## Discussion

### Principal Findings

In this study, we designed an innovative intervention based on a WAT with 2-stage goal-setting for daily steps and explored its efficacy in improving older adults’ PA and sarcopenia indicators. The total PA, LPA, sedentary time, BMI, WC, SMI, muscle strength, and physical performance of older adults who engaged in the 8-week intervention improved compared to those in the control group. Study results suggested that the WAT-based intervention with gradual goal-setting was beneficial for community-dwelling older adults’ PA and sarcopenia prevention. However, due to the cluster randomization, older adults in the experimental group were younger than those in the control group. The findings should be interpreted with caution.

A WAT with step-by-step goal setting improved older adults’ LPA and sedentary time but not MVPA after the 8-week intervention. The intervention was designed with daily steps as a goal, and with LPA as a targeted outcome for improvement, instead of MVPA. This is possibly why LPA was the only indicator that improved among multi-intensity types of PA. Previous studies also suggested that WAT-based interventions can improve participants’ PA, especially LPA. A previous randomized trial conducted a 6-week intervention with a pedometer with weekly goals. Their results also found that LPA improved but MVPA did not change after the intervention [33]. Another study conducted interventions with a WAT to record daily steps and PA with additional in-person health behavioral change techniques, including goal-setting, self-monitoring, building PA plans, and using social interactions, and the self-efficacy, daily steps, and the amount of PA of older adults who participated in the 3-month intervention improved; furthermore, participants’ PA level was maintained after the intervention [35].

WAT-based interventions provide behavioral change techniques to support and motivate older adults’ health self-management, PA engagement, and self-efficacy [19]. Results of a 3-arm randomized trial consisting of a control group, wearable group, and wearable group with goal-setting also found that the PA of older adults in the wearable group, which had a daily goal of 10,000 steps, had improved after 12 weeks [36]. Older adults are able to use WATs to understand physiological data, behavioral patterns, and health habits based on their personal records to promote awareness of their health conditions [23].

This study reinforced findings that WATs with 2-stage goal-setting for daily steps can improve older adults’ BMI and all sarcopenia indicators. Previous studies found similar results; PA reduced older adults’ sarcopenia risks. A previous randomized trial found that the physical performance and muscle strength of sedentary older adults engaged in a 2.6-year PA intervention with weekly goals improved compared to those who only received a health educational program [37]. Another randomized trial with a large sample size found that PA, physical performance, and muscle strength of older adults who received a 24-month walking intervention improved compared to those who only received discussion of a health-related topic [38]. Another study conducted a 6-month WAT-based intervention for sedentary community-dwelling older adults; PA, fear of falling, physical performance, and muscle mass of participants in the experimental group improved after the intervention [39].

This study found increased LPA and decreased MVPA after the intervention, but sarcopenia indicators improved nevertheless. In contrast, a previous study [40] suggested that there was a significant impact from reallocating sedentary time to at least LPA on reducing sarcopenia risks in older adults. Their findings indicated that engaging in LPA and MVPA was associated with a significantly lower sarcopenia risk. Reallocating more time to MVPA usually offers more protection for mitigating age-related muscle deterioration and promoting healthier aging [40].

Furthermore, both groups received identical weekly nutritional health education on healthy eating, which could have influenced their eating behaviors during the intervention and sarcopenia indicators. While sarcopenia indicators improved in the experimental group after the intervention, no such changes were observed in the control group. This suggests that the WAT-based intervention offered PA-promoting benefits. PA is an important protective factor for older adults that can be reinforced by goal-setting. Behavioral change techniques can be integrated into WAT-based interventions to prevent sarcopenia [7].

### Limitations

There are several limitations to this study. First, the major limitation is that it was a clustered trial with a small sample size that randomly assigned the community centers to either the control or intervention group, instead of using individual randomization. Variations in the characteristics of the community centers may have increased the risk of bias, as reflected in significant differences between them, particularly in participants’ ages (the experimental group mean age was 70.26 years, while the control group mean age was 74.26 years) and baseline scores. Although we used age and pretest scores as covariates for adjusting the ANCOVA and GEEs, the results should be interpreted with caution in future practice. Second, this study only used structured questionnaires to collect subjective data on PA and sedentary time. The accuracy might have been lower than if we had used objective PA measures, such as accelerometers and pedometers. In future studies, objective measures of PA and calories should be used during, before, and after the intervention, especially using WATs, as in this intervention. Third, the research team could not control users’ behavior and use patterns of the WATs. It is difficult to know the actual wearing time, how long they did not wear the device due to abnormal connections or other technical problems, or how many times they reached the goal of daily steps. The adherence rate might potentially be a major bias. Finally, the intervention was only conducted for 8 weeks before the community centers closed because of the COVID-19 pandemic. Improvements in PA outcomes and sarcopenia indicators could not absolutely be attributed to the WATs and 2-stage goal-setting.

### Implications

This study has implications for clinical application. Attention should be paid to sarcopenia risks in older adult populations. Health practitioners can advocate for older adults to adopt cost-efficient WATs to engage in walking. Due to advancements in technology, wearable devices feature increased accuracy without being invasive. The physiological data generated by WATs are expected to be an important reference for physicians and patients to make further medical decisions and health actions. The smartphone app may provide immediate health advice according to the data. WAT-based interventions are recommended as practical applications in older adults across different health care settings, such as long-term care facilities, daycare centers, and nursing homes.

This study also has implications for future research. Future studies can conduct WAT-based interventions to examine the health benefits of physiological indicators, such as blood pressure, blood glucose, and heart rate, across different older adult populations with chronic diseases. The goal of future WAT-based interventions could be to increase older adults’ MVPA, given its major impact on the development of sarcopenia. The duration of the intervention could be prolonged. Innovative and interesting wearable-based interventions can be designed according to the preferences of targeted populations. For increased adherence to WATs, the interventions could be integrated with different behavioral change techniques, including social support, rewards, reviews, competitions, and goal-setting for sedentary or exercise time. In addition, we did not have a platform to access personal data during the wearing period (eg, PA, heart rate, and calories). These data can be recorded to test the reliability and validity of the current WAT compared to other sensors, accelerometers, commercial WATs, and questionnaires.

### Conclusions

Smart wearable technologies are an innovative and efficient strategy for health behavioral modifications in older adult populations. Engaging in MVPA usually offers more protective benefits, while LPA by itself has a limited effect against developing sarcopenia. This study found that an 8-week intervention with a WAT and 2-stage goal-setting for daily steps improved older adults’ PA and sarcopenia indicators, including total PA, LPA, sedentary time, BMI, WC, muscle mass, muscle strength, and physical performance. This suggests that a WAT-based intervention with nutritional health education is feasible and efficient in older populations. However, the findings should be interpreted with caution when considering practical applications due to the age difference between the 2 groups. Commercial WATs with behavioral change techniques are recommended to promote older adults’ PA and prevent sarcopenia.

## Abbreviations

ANCOVA: analysis of covariance
GEE: generalized estimating equation
IPAQ-SF: International Physical Activity Questionnaire–Short Form
LPA: light-intensity physical activity
MD: mean difference
MET: metabolic equivalent of task
MVPA: moderate-to-vigorous-intensity physical activity
PA: physical activity
SMI: skeletal muscle index
WAT: wearable activity tracker
WC: waist circumference

## Data Availability

The datasets generated during and analyzed during the current study are available from the corresponding author upon reasonable request.
